# Surface Roughness and Wear Behavior of CAD/CAM Ceramics Against Vita Mark II

**DOI:** 10.1002/cre2.70174

**Published:** 2025-07-13

**Authors:** Amirhosein Habibnezhad, Gazaleh Ahmadi, Naeeme Naderi

**Affiliations:** ^1^ School of Dentistry Babol University of Medical Sciences Babol Mazandaran Province Iran

**Keywords:** ceramics, computer‐aided design, dental restoration wear, mechanical tests

## Abstract

**Objectives:**

This study aimed to assess the surface roughness (SR) and wear behavior of computer‐aided design/computer‐aided manufacturing (CAD/CAM) ceramics when compared to a feldspathic ceramic.

**Material and Methods:**

This in vitro study was conducted on three groups of CAD/CAM ceramic blocks (*n* = 10 each): a zirconia‐reinforced lithium silicate ceramic, a polymer‐infiltrated ceramic network, and a resin nanoceramic. Additionally, 40 feldspathic ceramic blocks and 10 bovine incisors were used as antagonist materials. Surface roughness was measured by profilometry before and after wear testing (49 N, 120,000 cycles, 30 cycles/min). Wear depth was quantified using 3D scanning and image analysis software. Data were analyzed using ANOVA and paired *t*‐tests (α = 0.05).

**Results:**

Before wear testing, the zirconia‐reinforced ceramic showed significantly higher SR than the other groups (*p* = 0.00). All groups exhibited a significant reduction in SR after testing (*p* = 0.00). Posttest SR varied significantly between most groups (*p* < 0.05), except between natural enamel and the resin nanoceramic (*p* = 0.986). Wear depth differed significantly among the materials (*p* < 0.001), with natural enamel showing the lowest wear, comparable only to the resin nanoceramic (*p* > 0.05).

**Conclusions:**

All tested CAD/CAM ceramics underwent substantial wear and SR reduction when opposed by feldspathic ceramic. The resin nanoceramic demonstrated the closest wear behavior to natural enamel and may be the most suitable restorative material for contact with feldspathic ceramics.

## 1 Introduction

The popularity of all‐ceramic restorations is on the rise due to the increased demand for esthetic restorations. Dental ceramics are currently used for the fabrication of inlays, onlays, crowns, and bridges. The majority of dental ceramic restorations may be fabricated conventionally or by the use of computer‐aided design/computer‐aided manufacturing (CAD/CAM) systems. In recent years, digital technology has gained increasing popularity in dentistry. The quality of restorations fabricated by computerized techniques is favorably high (Turker and Kursoglu 2021). However, despite ideal properties such as optimal esthetics, matching the natural tooth color, favorable mechanical properties, chemical stability, and optimal biocompatibility, they have drawbacks such as causing occlusal wear of the opposing teeth/restorations, which can lead to the loss of anatomical form and esthetic problems, among others (Elsaka 2015; Someya et al. 2023). Thus, wear behavior should be taken into account in the selection of restorative materials with long‐term clinical service (Ozkir et al. 2022).

Despite the high accuracy of intraoral scanners and CAD/CAM milling machines, the final restorations may need adjustments, which should be followed by a subsequent final finishing. However, the finished surface often remains rough, enhancing plaque accumulation and staining, and causing wear of the opposing teeth and restorations (Borgia et al. 2019).

The CAD/CAM process can be divided into three phases of data collection, indirect restoration design, and restoration fabrication (Miyazaki et al. 2009). Each step requires a certain level of precision. Also, each step is independent of the other steps; although the scans determine the digital model, and enable designing of indirect restorations (van Noort 2012).

Occlusal wear of the teeth and restorations is a complex multifactorial process that depends on several biological, mechanical, chemical, and frictional factors. The depth of wear depends on a number of factors, such as muscle strength, presence/absence of saliva, oral habits, and type of restorative material. Correct selection of restorative materials is important to ensure occlusal harmony and normal masticatory function (Anusavice et al. 2012). The depth of enamel wear under masticatory forces is reportedly 20 to 40 µm per year (Spear and Holloway 2008).

Ideally, wear of restorative materials should be comparable to natural enamel wear. Nonetheless, some restorative materials have a different wear behavior than enamel, and can cause a different depth of wear in the opposing teeth/restorations (Spear and Holloway 2008). Highly abrasive dental materials can damage the occlusal surface, change the functional path of occlusal movements, and lead to the loss of anterior guidance and esthetic problems (Suliman et al. 1993).

Metal‐ceramic restorations are increasingly replaced with all‐ceramic restorations due to their suboptimal esthetics and translucency, poor metal‐porcelain bond, and incompatible properties of metal and porcelain (De Angelis et al. 2020; Fabianelli et al. 2010; Kelsey et al. 2000; Noel and Mitchell 1997; Ozcan and Niedermeier 2002; Park et al. 2014).

Vita Mark II remains one of the most widely used feldspathic ceramics in esthetic and minimally invasive restorations, accounting for over 25% of single‐unit crowns in Europe and North America (Fabianell et al. 2010). It has long‐term survival rates (> 95% at 5 years) and it has distinct microstructural features that influence wear characteristics (De Angelis et al. 2020). Additionally, Vita Mark II ceramic was among the first CAD/CAM ceramics introduced (Park et al. 2014).

Considering the limited information available about the surface roughness (SR) and wear behavior of different ceramics against VITA Mark II ceramic, this study aimed to assess the SR and wear behavior of CAD/CAM ceramics against Vita Mark II.

## 2 Materials and Methods

This in vitro experimental study was conducted on 10 Vita Suprinity, 10 Vita Enamic, and 10 Lava Ultimate CAD/CAM ceramic blocks, 40 Vita Mark II ceramic blocks, and 10 bovine incisors (as a control group) (Table 1).

**Table 1:** CAD‐CAM ceramics evaluated in this study.

Ceramic	Manufacturer	Composition
VITABLOCKS MARK II (18 × 14 × 12)	VITA Zahnfabrik Germany	Monolithic blocks
Vita Enamic	VITA Zahnfabrik Germany	86% feldspathic ceramic network and 14% resin
VITA Suprinity	VITA Zahnfabrik, Germany	Zirconia‐reinforced lithium silicate glass ceramic
Lava Ultimate	Lava Ultimate; 3 M ESPE, MN	80 wt% nano‐ceramic (zirconia and silica) and resin network

Bovine incisors share similar enamel prism structure and hardness (mean Vickers hardness difference < 5%) with human enamel (Yassen et al. 2011). It has strong correlation (*R*² > 0.9) between bovine‐based and human enamel wear outcomes under identical loading (Wang et al. 2021).

The study protocol was approved by the ethics committee of the university (IR.MUBABOL.HRI.REC.1402.054).

### 2.1 Sample Size

The sample size was calculated according to previous studies (Someya et al. 2023; Turker and Kursoglu 2021) using the sample size calculation formula as follows:

$$\begin{array}{l} n=\frac{{\left(z_{1-\alpha /2}+z_{1-\beta }\right)}^{2}\left[s_{1}^{2}+s_{2}^{2}\right]}{{\left(\mu_{1}-\mu_{2}\right)}^{2}}\\ n=\frac{8\times \left(0.63^{2}+0.25^{2}\right)}{{\left(1.68-2.45\right)}^{2}}\\ =6.19 \end{array}$$

### 2.2 Specimen Preparation

All blocks were sectioned in a precision sectioning machine (Nemo Fanavaran Pars, Mashhad, Iran) to obtain 10 specimens from each ceramic block, measuring 5 × 5 mm with 2 mm thickness. The specimens were polished with a ceramic polishing kit (Drendel Zweiling, Switzerland), which had three polishing discs. Ten enamel specimens were also obtained from the labial surface enamel of bovine incisors using a diamond disc and measured 5 × 5 mm with a 2 mm thickness. The specimens were polished with silicon carbide abrasive papers. All enamel and ceramic specimens were mounted in similar acrylic resin molds, and polished with 200‐, 400‐, 600‐, and 1200‐grit silicon carbide abrasive papers for 1 min (Amer et al. 2015; Lauvahutanon et al. 2015).

### 2.3 Measurement of SR

The SR of all specimens was measured before and after the wear test by using a 3D profilometer (Nemo Fanavaran Pars, Mashhad, Iran), and the results were reported quantitatively (Elsaka 2015). One specimen from each group was also randomly selected for inspection under an atomic force microscope (AFM; Nanosurface, Switzerland). The SR of each specimen was measured at three different points with a cut‐off of 0.8 mm, and the mean of the three values was calculated and reported as the SR of the respective specimen.

### 2.4 Wear Test

**Figure 1:**
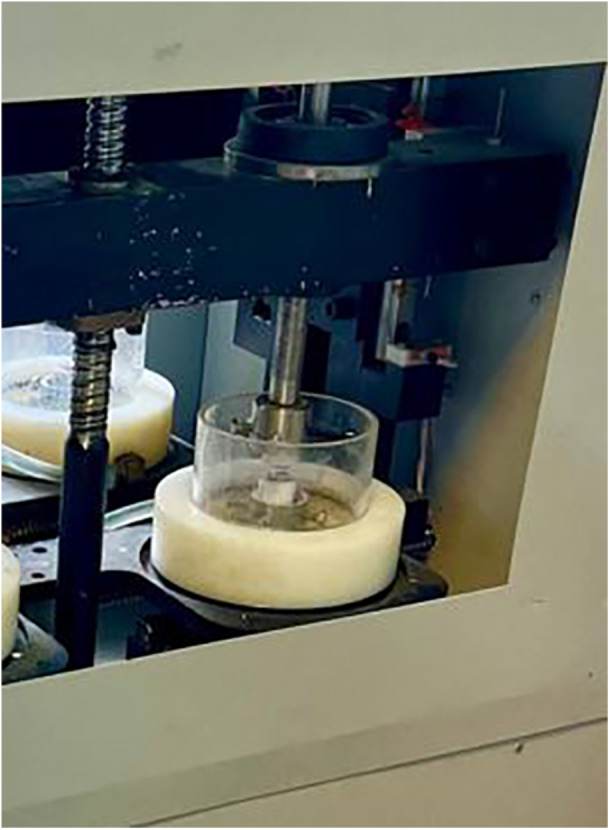
Wear test in a chewing simulator.

A chewing simulator (Nemo Fanavaran Pars, Mashhad, Iran) was used for the wear test (Figure 1). It applied 49 N load equal to 5 kg to the ceramic specimen. The ceramic specimen moved downward, and upon contacting the opposing specimen, it moved horizontally by 2 mm (van Noort 2012). VITA Mark II feldspathic ceramic was used in the mandibular component as the antagonist while other specimens were placed in the maxillary component of the chewing simulator. Next, the maxillary component was spaced from the opposing ceramic by 3 mm, and the masticatory cycle was repeated for 120,000 cycles (corresponding to 6 months of mastication in the oral cavity) (van Noort 2012) at a speed of 30 cycles/minute in presence of toothpaste (Crest 7) according to Lauvahutanon et al. (2015).

### 2.5 Depth of Wear Assessment

After measuring the SR of the specimens for the second time after the wear test, their surface was scanned by a 3D scanner (I3Dscan, Germany), and the depth of wear in each group was calculated using Photoshop Ps software.

### 2.6 Statistical Analysis

Data were analyzed using SPSS version 24 (SPSS Inc., IL, USA). Normal distribution of data was confirmed by the Kolmogorov–Smirnov test. Thus, comparisons were made by ANOVA and paired sample *t*‐test at 0.05 level of significance.

## 3 Results

### 3.1 SR

Table 2 shows the SR of the specimens in the four groups before and after the wear test. As shown, the SR of the specimens significantly decreased after the wear test in all four groups (*p* = 0.00).

**Table 2:** SR of specimens in the four groups before and after the wear test.

Group	SR	Mean	Std. deviation	*p*‐value*
Enamel	Before	154.377	0.54838	0.00
	After	11.3305	0.45490	
	Difference	143.0472	0.69203	
Enamic	Before	154.2244	0.32809	0.00
	After	143.0876	0.86885	
	Difference	11.1368	0.95063	
Suprinity	Before	156.1229	1.43012	0.00
	After	26.1545	1.96551	
	Difference	129.9684	2.03014	
Ultimate	Before	153.3385	0.99113	0.00
	After	11.5143	1.02381	
	Difference	141.8242	1.11842	

* ANOVA.

Comparison of the SR of the four groups before the wear test (Table 3) revealed that the SR of Suprinity was significantly higher than that of all other groups (*p* = 0.001). No other significant differences were found (*p* > 0.05).

**Table 3:** Pairwise comparisons of SR of the four groups before the wear test (*n* = 10).

Study groups compared			Mean	Std. Deviation	*p*‐value*
	Enamel	Enamic	0.15330	0.41448	0.982
		Suprinity	−1.74520	0.41448	0.001
		Ultimate	1.03920	0.41448	0.076
SR before the wear test	Enamic	Enamel	−0.15330	0.41448	0.982
		Suprinity	−1.89850	0.41448	0.000
		Ultimate	0.88590	0.41448	0.161
	Suprinity	Enamel	1.74520	0.41448	0.001
		Enamic	1.89850	0.41448	0.000
		Ultimate	2.78440	0.41448	0.000
	Ultimate	Enamel	−1.03920	0.41448	0.076
		Enamic	−0.88590	0.41448	0.161
		Suprinity	−2.78440	0.41448	0.000

* Tukey HSD test.

After the wear test (Table 4), significant differences were found between all other groups (*p* < 0.05), except between natural enamel and Ultimate ceramic (*p* = 0.986).

**Table 4:** Pairwise comparisons of SR of the four groups after the wear test (*n* = 10).

Study groups			Mean	Std. Deviation	*P*‐value*
	Enamel	Enamic	−131.75710	0.54191	0.00
		Suprinity	−14.82400	0.54191	0.000
		Ultimate	−0.18380	0.54191	0.986
SR after the wear test	Enamic	Enamel	131.75710	0.54191	000
		Suprinity	116.93310	0.54191	000
		Ultimate	131.57330	0.54191	000
	Suprinity	Enamel	14.82400	0.54191	000
		Enamic	−116.93310	0.54191	000
		Ultimate	14.64020	0.54191	000
	Ultimate	Enamel	0.18380	0.54191	0.986
		Enamic	131.57330	0.54191	000
		Suprinity	−14.64020	0.54191	000

* Tukey HSD test.

Comparison of the mean change in SR after the wear test compared with before among the four groups (Table 5) revealed significant differences between all groups except between natural enamel and Ultimate ceramic (*p* = 0.171).

**Table 5:** Comparison of the mean change in SR after the wear test compared with before among the four groups (*n* = 10).

Study groups			Mean	Std. Deviation	*p*‐value*
	Enamel	Enamic	131.91040	0.58116	0.00
		Suprinity	13.07880	0.58116	0.00
		Ultimate	1.22300	0.58116	0.171
Mean change in SR after the wear test compared with before	Enamic	Enamel	−131.91040	0.58116	0.00
		Suprinity	−118.83160	0.58116	0.00
		Ultimate	−130.68740	0.58116	0.00
	Suprinity	Enamel	−13.07880	0.58116	0.00
		Enamic	118.83160	0.58116	0.00
		Ultimate	−11.85580	0.58116	0.00
	Ultimate	Enamel	−1.22300	0.58116	0.171
		Enamic	130.68740	0.58116	0.00
		Suprinity	11.85580	0.58116	0.00

* Tukey HSD test.

### 3.2 Depth of Wear

A significant difference was found in the depth of wear among the four groups (*p* < 0.001, Table 6). Pairwise comparisons (Table 6) revealed significant differences in depth of wear between Enamic and Ultimate (*p* < 0.001), and also between Suprinity and Ultimate (*p* < 0.001). Also, the depth of wear of enamel was significantly lower than that of all other ceramics (*p* < 0.001), except for Ultimate (*p* > 0.05).

**Table 6:** Depth of wear (mm) in the four groups (*n* = 10).

Group	Mean ± std. deviation
Enamel	^A^0.004 ± 0.056
Enamic	^B^0.006 ± 0.082
Suprinity	^B^0.005 ± 0.09
Ultimate	^A^0.018 ± 0.06
*p* < 0.001*	

* ANOVA: similar uppercase letters indicate the absence of a significant difference in depth of wear.

### 3.3 AFM

AFM was used to support and visualize surface texture differences observed after the wear test.

**Figure 2:**
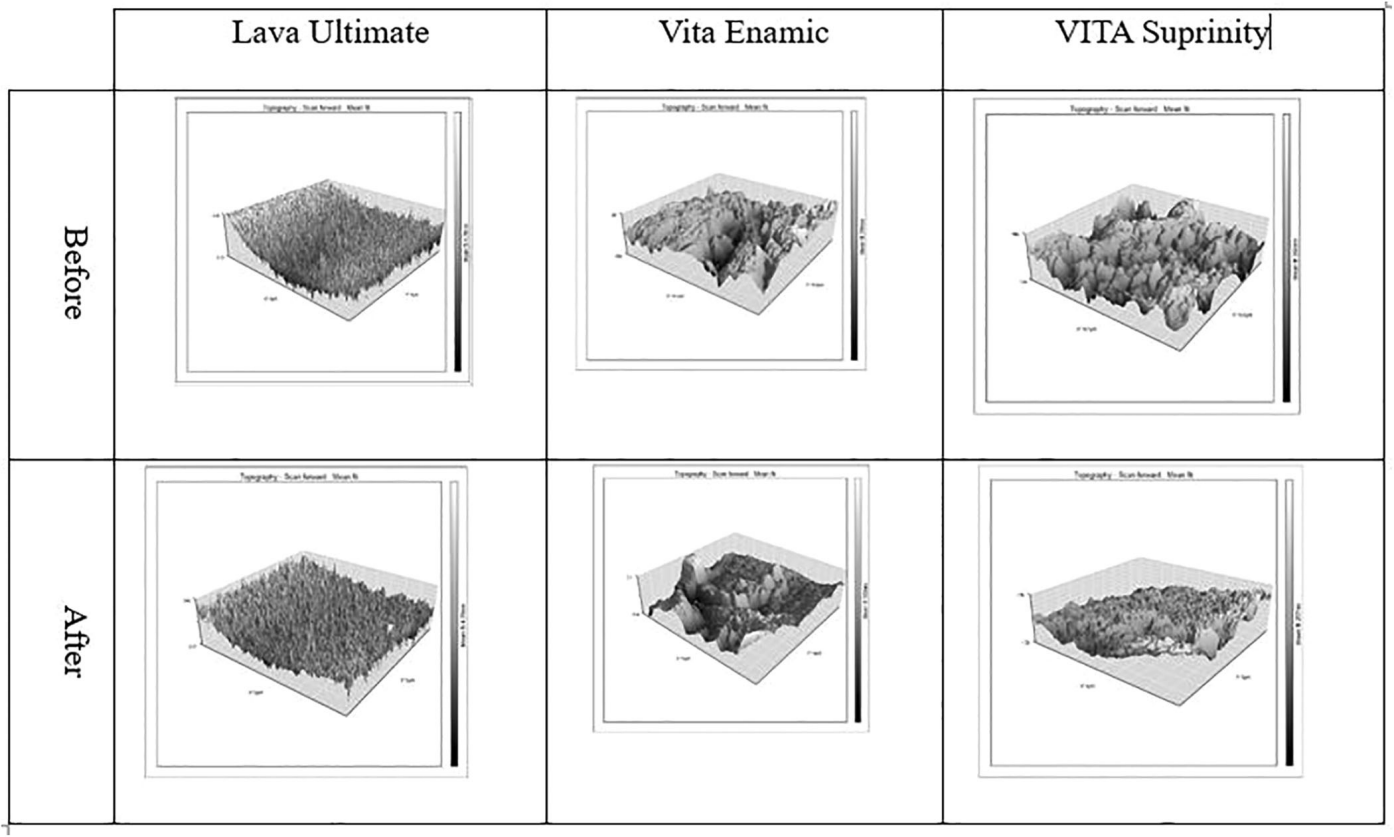
SR of ceramics according to AFM assessment before and after the wear test.

The SR of Suprinity and Ultimate decreased after the wear test as shown on AFM micrographs; however, changes in Enamic were not clearly noticeable (Figure 2).

## 4 Discussion

This study assessed the SR and wear behavior of CAD/CAM ceramics against Vita Mark II. The results showed that the SR of the specimens significantly decreased after the wear test in all four groups. The SR of Suprinity was significantly higher than that of other ceramics before the wear test. No other significant differences were found. The present results were in agreement with the findings of Naumova et al. (2017), who measured the SR of Vita Enamic, Lava Ultimate, and Vita Suprinity before and after the wear test and showed that Suprinity had the highest SR before the wear test.

Since both Enamic and Ultimate ceramics have a resin matrix, they have a combination of physical and mechanical properties of ceramics, and flexural and limited wear properties of composite resins (Çelik et al. 2018). They have a flexural strength and modulus of elasticity similar to those of tooth structure and a hardness lower than that of other ceramics (Albero et al. 2015). Thus, due to their higher resemblance to tooth structure, they cause less wear of natural enamel than other ceramics (Özarslan et al. 2016). According to Wang et al. (2012), materials with a modulus of elasticity different from that of enamel cause stress at the enamel surface and its subsequent wear. Therefore, the lack of a significant difference in baseline SR between most ceramics and enamel is due to their similar structure to enamel structure.

Surface roughness is a critical factor influencing the clinical performance and longevity of dental restorations (Naumova et al. 2017). The occlusal SR affects plaque accumulation, restoration wear, staining, color match, and wear of the opposing teeth/restorations (Naumova et al. 2017). Submicron alterations in surface texture can significantly affect antagonist wear rates and plaque accumulation. Increased surface roughness has been associated with higher bacterial adhesion and biofilm formation, which can lead to secondary caries and periodontal issues. Moreover, rougher surfaces contribute to greater wear on opposing dentition, potentially compromising the integrity of both the restoration and the antagonist tooth (Quirynen and Bollen 1995).

A number of factors affect the SR and wear of restorations and the opposing natural teeth, such as finishing and polishing of restorations, glazing quality, flexural strength, modulus of elasticity, and solubility of ceramics, chewing frequency, masticatory forces, duration of contact, sliding movements, hardness of foods, saliva pH, gastrointestinal diseases, and toothbrushing (Naumova et al. 2017). Phase, type, dispersion, and shape of crystal grains in ceramics also affect their wear properties. Vita Mark II ceramic has angulated and scale‐like crystals as shown by scanning electron microscopic assessments.

Clinical studies have demonstrated that restorations with higher surface roughness exhibit increased failure rates over time. These findings underscore the importance of achieving and maintaining a smooth surface finish on restorations. Our results support the implementation of routine intraoral polishing protocols for Vita Mark II restorations to enhance their longevity and clinical success (Bollenl et al. 1997, Söderholm et al. 1990).

In a previous study, the SR of all ceramics significantly decreased after the wear test against Vita Mark II felspathic ceramic, except for Vita Enamic, in which, the reduction was not significant (Someya et al. 2023). Enamic showed only a slight reduction in surface roughness after the wear test (Ra: 154 µm → 143 µm), in contrast to other ceramics, which demonstrated larger reductions. We believe this can be explained by the distinct dual‐network structure of Enamic, which combines a feldspathic ceramic network (86%) with an interpenetrating polymer phase (14%). This hybrid composition results in mechanical behavior that differs significantly from monolithic ceramics: (1) Dual‐network structure (86% ceramic, 14% polymer): VITA ENAMIC is the first hybrid dental ceramic in the world with a dual‐network structure. The dominant ceramic network (86% by weight) is strengthened by a polymer network (14% by weight), with both networks fully integrated (Güngör et al. 2016). (2) Elastic modulus (~29–30 GPa): VITA Enamic exhibits an elastic modulus ranging from approximately 21.5–37.95 GPa, with an average around 30 GPa, aligning closely with the properties of natural dentin (Ceren 2021; Lan 2021). (3) Polymer smear layer effect on Ra measurements: The polymer component in VITA Enamic tends to smear during wear, potentially masking true material loss and artificially elevating surface roughness (Ra) measurements. (4) Similarity in composition to Vita Mark II: Both VITA Enamic and Vita Mark II are feldspathic‐based ceramics, which may contribute to reduced abrasion efficiency when interacting with each other (Güngör et al. 2016).

Comparison of the SR of different groups before and after the wear test revealed significant differences between all groups except between enamel and Ultimate ceramic. Daryakenari et al. (2019) evaluated the SR of different types of glazed and polished ceramics before and after the wear test, and reported that the SR of glazed Vita Mark II after the wear test was significantly lower than other groups. It should be noted that Ultimate ceramics showed the greatest change in SR in the wear process. High SR of Ultimate ceramic may be due to large size of its crystals. This finding was in contrast to the results of Moörmann (2006), who showed that Ex heat press ceramic caused a higher Ra value in the opposing tooth. This difference in the results of the two studies may be attributed to the use of different ceramic types and greater structural homogeneity of CAD/CAM ceramics compared with heat press ceramics.

Assessment of wear behavior of dental restorative materials is clinically important since it can affect the restoration appearance, change inter‐arch relationship due to tooth movements, decrease the vertical height of occlusion, adversely affect the masticatory function, and cause eventual muscle fatigue (Ekfeldt and Karlsson 1996). In the present study, all ceramic specimens experienced wear after the wear test due to the structure of Vita Mark II. It also caused the wear of natural enamel. Considering the highly similar physical properties of Ultimate ceramic and natural enamel, the depth of wear of Ultimate ceramic against Vita Mark II was almost similar to the depth of wear of natural enamel (Albero et al. 2015). Also, the depth of wear of enamel was significantly lower than that of other ceramics, except for Ultimate, which had no significant difference with enamel in wear depth. A previous study demonstrated the highest depth of wear in natural enamel against glazed Vita Mark II (Elsaka 2015). Naumova et al. (2017) revealed a significant difference in depth of wear and volume loss of different ceramics, and the highest volume loss was reported for Ultimate followed by Suprinity while the smallest volume loss was recorded for Enamic, which was different from the present results. This difference may be due to the use of a different antagonist, i.e., Vita Mark II in the present study, which has an abrasive nature, while they used natural teeth as an antagonist in their study (Naumova et al. 2017). Another study reported the greatest depth of wear in Filtek Supreme XTE composite resin and Plus HRi Enamel against a CAD/CAM Y‐TZP ceramic (De Angelis et al. 2020). Shimane et al. (2010) discussed that between two materials, the softer material wears more easily than the harder material (Shimane et al. 2010). Thus, the wear of composite resins against ceramics would be greater than that of ceramics.

In the present study, increased SR resulted in greater wear of the opposing enamel. Severe enamel wear can lead to loss of centric contacts, change the vertical facial height, alter the functional paths of occlusion, and cause masticatory muscle fatigue (De Gee et al. 1986; DeLong et al. 1989; Gallegos and Nicholls 1988). Thus, the wear behavior of dental restorative materials is an important parameter that should be taken into account when selecting a restorative material. The wear behavior of restorative materials should be close to that of natural enamel as much as possible. Some previous studies used SR values to estimate the wear properties and depth of wear (Janyavula et al. 2013; Oh et al. 2002). Turker and Kursoglu (2021) demonstrated a smaller volume loss in IPS e.max ZirCAD than other ceramics. Also, cuspal wear was greater against Celtra Duo than GC Cerasmart. According to their study, polymer‐infiltrated ceramic network materials and zirconia‐reinforced lithium silicate ceramic (Celtra Duo) showed greater wear (ceramic volume loss) against the opposing enamel, unlike zirconia ceramic with a homogenous structure. Moreover, GC cerasmart nano‐ceramic block caused minimal enamel wear due to its flexible structure (Turker and Kursoglu 2021). Someya et al. (2023) indicated that IPS e.max CAD and Celtra Duo ceramics experienced greater volumetric wear due to their crystalline structure with a large grain size. Furthermore, Celtra Duo caused the highest wear in the opposing enamel. They found no significant correlation between the mechanical properties of ceramics such as their hardness and 3‐point flexural strength with their volumetric wear. Instead, the type of glass ceramic, and the size and shape of crystal grains, dictated the wear behavior of ceramics (Someya et al. 2023).

This study had an in vitro design. Thus, the generalization of results to the clinical setting must be done with caution. Higher number of chewing cycles are recommended in future studies to better simulate the clinical setting. Further studies are also required to assess the wear behavior of different composite resins.

## 5 Conclusion

These findings emphasize that Vita Mark II, despite being a feldspathic ceramic, exhibits distinctive wear interactions with other CAD/CAM materials. While most tested materials showed a significant reduction in surface roughness after wear, Vita Enamic demonstrated no statistically significant change, likely due to its polymer–ceramic hybrid structure. The observed differences in SR have direct clinical relevance, influencing both the longevity of restorations and the preservation of opposing dentition. These insights may support clinicians in material selection and underline the importance of appropriate finishing and polishing protocols to enhance long‐term clinical outcomes.

## Author Contributions

Study concept and design: Naeeme Naderi. Acquisition of data: Gazaleh Ahmadi. Analysis and interpretation of data: Naeeme Naderi. Drafting of the manuscript: Amirhosein Habibnezhad. Critical revision of the manuscript for important intellectual content: Amirhosein Habibnezhad. Statistical analysis: Gazaleh Ahmadi. Administrative, technical, and material support: Naeeme Naderi. Study supervision: Gazaleh Ahmadi.

## Conflicts of Interest

The authors declare no conflicts of interest.

## Data Availability

The data that support the findings of this study are available from the corresponding author upon reasonable request.
